# Surface-modified three-dimensional graphene nanosheets as a stationary phase for chromatographic separation of chiral drugs

**DOI:** 10.1038/s41598-018-33075-w

**Published:** 2018-10-03

**Authors:** Lindsay Candelaria, Liliya V. Frolova, Brian M. Kowalski, Kateryna Artyushkova, Alexey Serov, Nikolai G. Kalugin

**Affiliations:** 1 Department of Materials and Metallurgical Engineering, New Mexico Tech, Socorro, NM 87801 USA; 2 Department of Chemistry, New Mexico Tech, Socorro, NM 87801 USA; 3 Department of Biological and Chemical Engineering, University of New Mexico, Albuquerque, NM 87131 USA; 4 Pajarito Powder, LLC, 3600 Osuna Rd NE, Suite 309, Albuquerque, NM 87109 USA

## Abstract

Carbon-based stationary phases for chromatographic separation have been commercially available since the 1980s. Porous graphitic carbon liquid chromatography columns are known to be highly resistant to aggressive mobile phases and extreme pH values of solvents and eluents, an important advantage compared to conventional silica-based alternatives. In our work, we demonstrate a new variant of carbon-based stationary phases for liquid chromatography, specifically developed for chiral separation. Mesoporous three-dimensional graphene nanosheets (3D GNS), functionalized with tetracyanoethylene oxide (TCNEO) and (S)-(+)-2-pyrrolidinemethanol, demonstrate pharmaceutical-grade chiral separation of model ibuprofen and thalidomide racemic mixtures when used as Chiral Stationary Phases (CSPs), with performance parameters comparable to currently commercially available CSPs. Simple covalent attachment of functionalization groups to the surface of mesoporous three-dimensional graphene nanosheets makes these carbon-based CSPs chemically stable and up to an order of magnitude less expensive than standard silica-based analogues.

## Introduction

More than half of the drugs currently in use are chiral compounds, and nearly 90% of them are marketed as racemic mixtures consisting of an equimolar amount of two enantiomers. Although they have the same chemical structure, enantiomers of chiral drugs exhibit dramatic differences in their pharmaceutical effects, such as pharmacokinetics, metabolism, and toxicity^1^. Very frequently, only one of the enantiomers provides the desired medicinal effect. Elimination of the unwanted enantiomer from utilization in pharmaceutical formulations requires chiral separation and analysis of racemic drugs in the industry and clinic^2^.

**Figure 1:**
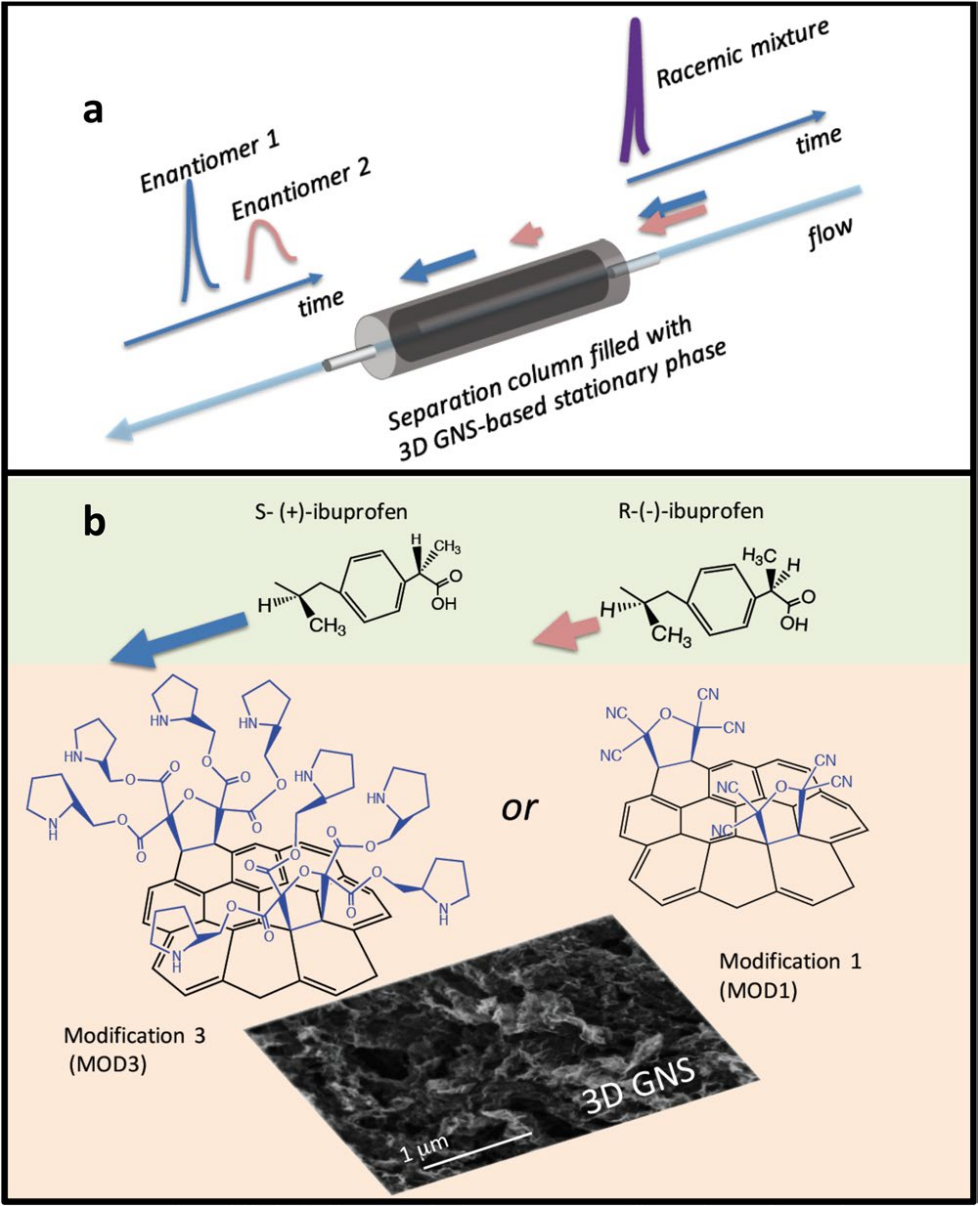
(**a**) The principle of HPLC chiral separation: a flow of dissolved chiral drug (racemic mixture containing both enantiomers) passes a chromatographic column filled with chiral stationary phase material (a 3D GNS-based material in our case). Due to a difference of retention time for enantiomers of opposite symmetry, one enantiomer passes the column faster than the other, so the enantiomers are separated. (**b**) Chromatographic separation of a chiral drug (in this case, ibuprofen in solution) with MOD3 or MOD1 3D GNS-containing stationary phase material. The colors of backgrounds indicate the mobile phase (light olive) and the stationary phase parts (cream color) of the separation process. In our experiments, positive optical rotation angle-enantiomers of ibuprofen and thalidomide passed through 3D GNS-containing separation columns faster than the corresponding negative rotation angle-enantiomers. The bottom part of the figure illustrates both chemical structure and morphology (SEM image) of mesoporous modified 3D GNS materials.

Common chiral separation techniques are gas and liquid chromatography (GC, LC) and capillary electrophoresis (CE)^3^. All of these techniques are based on an enantioselective interaction between the enantiomers and the chiral selectors. Separation usually takes place during the transport of drug molecules through separation-supporting material, and is quantified based on the difference in retention time for different enantiomers (Fig. 1a). High performance liquid chromatography (HPLC) is the most frequently used method because of its relative simplicity and efficiency^3^. Capillary electrophoresis, the other robust chiral separation technique, is limited in industrial applications due to relatively small throughput^3^.

In HPLC systems, enantiomers can be separated using a chiral selector either immobilized on the stationary phase or dissolved in the mobile phase. Currently, the most common approach used for HPLC chiral separation is the utilization of stationary phases made of silica-based materials modified with chiral selectors (for example cellulose molecules or cyclodextrins). Such coated-type CSPs demonstrate good separation capabilities, but insufficient chemical stability, allowing only a rather limited number of separation cycles prior to destruction of the column and limiting the range of solvents for the mobile phase^3,4^. Another disadvantage of silica-packed columns is their high cost. Chemical stability of silica-based columns was significantly improved in silica gel CSPs with immobilized chiral selectors, but the complexity of fabrication makes these CSPs even less affordable^4^.

In the 1970s, in light of the drawbacks associated with silica-based stationary phases, specifically related to the fragility and instability of the bonded ligands, attention shifted to carbon as a potential alternative^5,6^. The idea of covering aggregates of graphitized carbon black or silica particles with pyrolytic carbon stimulated the development of porous glassy carbon^7–10^. Another significant achievement was the demonstration of porous graphitic carbon (PGC)^11,12^. Good separation results were achieved using PGC (commercial Hypercarb®, Carbon-500®, BTR carbon materials), active carbons, graphitized carbon black, glassy carbon, and colloid-imprinted carbon^5,13–17^. PGC-based stationary phases are nearly insensitive to aggressive mobile phases and operating conditions; in particular, they are highly resistant to extreme pH values^5^ and can be employed between pH 0 and 14. They also resist high temperatures, making them suitable for high temperature liquid chromatography^18^. All of these products and materials were in general not designed for chiral separation, and silica-based stationary phase materials continue to be the primary tools for such applications. While there were successful attempts of using PGC in chiral ion-pair chromatography^19–22^, and of employing electrosorption-based modification of porous graphitic carbon for chromatographic chiral separation^23^, no such solutions gained widespread adoption.

The discovery of carbon nanotubes (CNT) triggered an abundant increase of activity in the development of carbon-based separators for chromatography. CNT-based stationary phases demonstrated promising results in chiral separation, especially using chemically-modified (functionalized) nanotubes^24–27^. Some authors also discussed ideas of using the intrinsic chirality of CNTs in separation^28^. Factors limiting a wider use of CNT-based stationary phases include a) the mobility of nanotubes, requiring the involvement of other materials, such as organic polymers, to form a stable matrix and hold nanotubes in place, and b) the high price of carbon nanotubes.

The most recent spike of interest in carbon-based chiral separators is related to the implementation of another allotropic form of carbon – graphene – and related materials, such as graphene oxide (GO) and reduced graphene oxide (rGO)^29^. The works^30–34^ report on chiral separation and chiral sensing attempts utilizing different variants of electrochemical techniques. In these works, graphene oxide or rGO was used as one of components of modified electrodes or as a part of (quasi)stationary phases for capillary electrochromatography. In the work ref.^35^, a beta-cyclodextrin- modified GO in liquid phase was used for chiral recognition in cyclic voltammetry and electrochemical impedance spectroscopy. An interesting recent approach in chiral separation techniques involves the creation of separation membranes made out of chemically functionalized graphene-based materials. Hauser *et al*. recently discussed the idea of using holes in planar graphene for transport of chiral compounds across graphene layers^36^. The authors demonstrated models showing the possible function of chiral molecule-functionalized holes in graphene as efficient “gatekeepers” for the separation of chiral molecules traveling across graphene layers^36^. Another work in this field reports the usage of L-glutamic acid-functionalized GO membranes for the separation of 3,4-dihydroxy-phenylalanines^37^. A promising direction in the development of chiral separation techniques is the use of graphene-based mesoporous materials^38–40^. Fuchs *et al*. tested the idea of making intrinsically chiral mesoporous carbon for use in enantioselective chemistry, synthesized via carbonization of chiral ionic liquids^38^. The works^39,40^ report on 90–95% separation selectivity of D-amino acids on imine formed by reaction with (S)-alanine racemase chiral analogue ((S)-ARCA) adsorbed on inner surfaces of pores in meso/macroporous monolithic carbon.

Despite this significant progress, no reports have so far demonstrated CNT or graphene-based CSPs for use in HPLC that are fully competitive with commercial silica-based immobilized CSPs. The reported carbon-based CSPs using chiral CNT-doped organic polymer monoliths are complex in fabrication. They have limitations related to insufficient control of organic monolith porosity during fabrication, the effect of swelling of organic monoliths in organic solvents, as well as complications and costs related to the need for small diameter (tens-hundreds of μm) capillary tubes and dramatically low possible flow rates (μl/min or less) through the separation columns^25,41^. The demonstrated chiral separators utilizing graphene or graphene oxide have a variety of limitations: they either use weakly attached (adsorbed) chiral molecules^29–35^ and, as a result, cannot be stable in many important organic solvents, or they completely adsorb and block one of the sample enantiomers during separation, being specifically designed for such a non-chromatographic type of separation^39,40^.

In the present work, we demonstrate the fabrication of competitive, cost- and performance-efficient graphene-based CSPs, as well as the production of the corresponding separation columns compatible with standard commercial HPLC chromatographs. We developed the technology that allows the production of a mesoporous 3D GNS-based material with controllable porosity, surface chemistry and liquid transport properties. Simple one- or three-step reactions that covalently attach functional groups via C-C bonds to the surface of mesoporous three-dimensional graphene nanosheets make these carbon-based CSPs chemically stable, versatile, and at least several times cheaper that the currently used silica-based industrial chiral separation columns.

## Results

The following two variants of modified mesoporous 3D GNS stationary phase materials were synthesized: 3DGNS material surface functionalized with tetracyanoethylene oxide (TCNEO)- modification 1 (MOD1) and 3DGNS material functionalized in three synthetic steps starting from MOD1, subsequent hydrolysis and final esterification with (S)-(+)-pyrrolidinemethanol- Modification 3 (MOD3). The details of the synthesis procedure are described in Methods. The concept of chromatographic chiral separation utilizing synthesized materials is illustrated in Fig. 1. In our separation experiments, two important chiral drugs were used: ibuprofen ((±)-2-(4-Isobutylphenyl)propanoic acid) (Sigma Aldrich 51146-56-6, 15687-27-1) and thalidomide ((±)-2-(2, 6-Dioxo-3-piperidinyl)-1H-isoindole-1, 3(2H)-dione) (Sigma Aldrich 1652500). In our experiments, we used commercial S and R enantiomers of ibuprofen as well as commercial racemic ibuprofen and thalidomide.

The separation experiments were done using flash chromatography in pipette columns, and HPLC in standard configuration, utilizing columns filled with 3D GNS-based stationary phase materials. Flash chromatography pipette experiments allowed the collection of sufficient amounts of separated enantiomers of ibuprofen and thalidomide and utilization of polarimetry for the characterization of separation capabilities of synthesized 3D GNS-based stationary phases. The detailed description of flash chromatography separation and polarimetry results is provided in the Supplementary Information.

**Figure 2:**
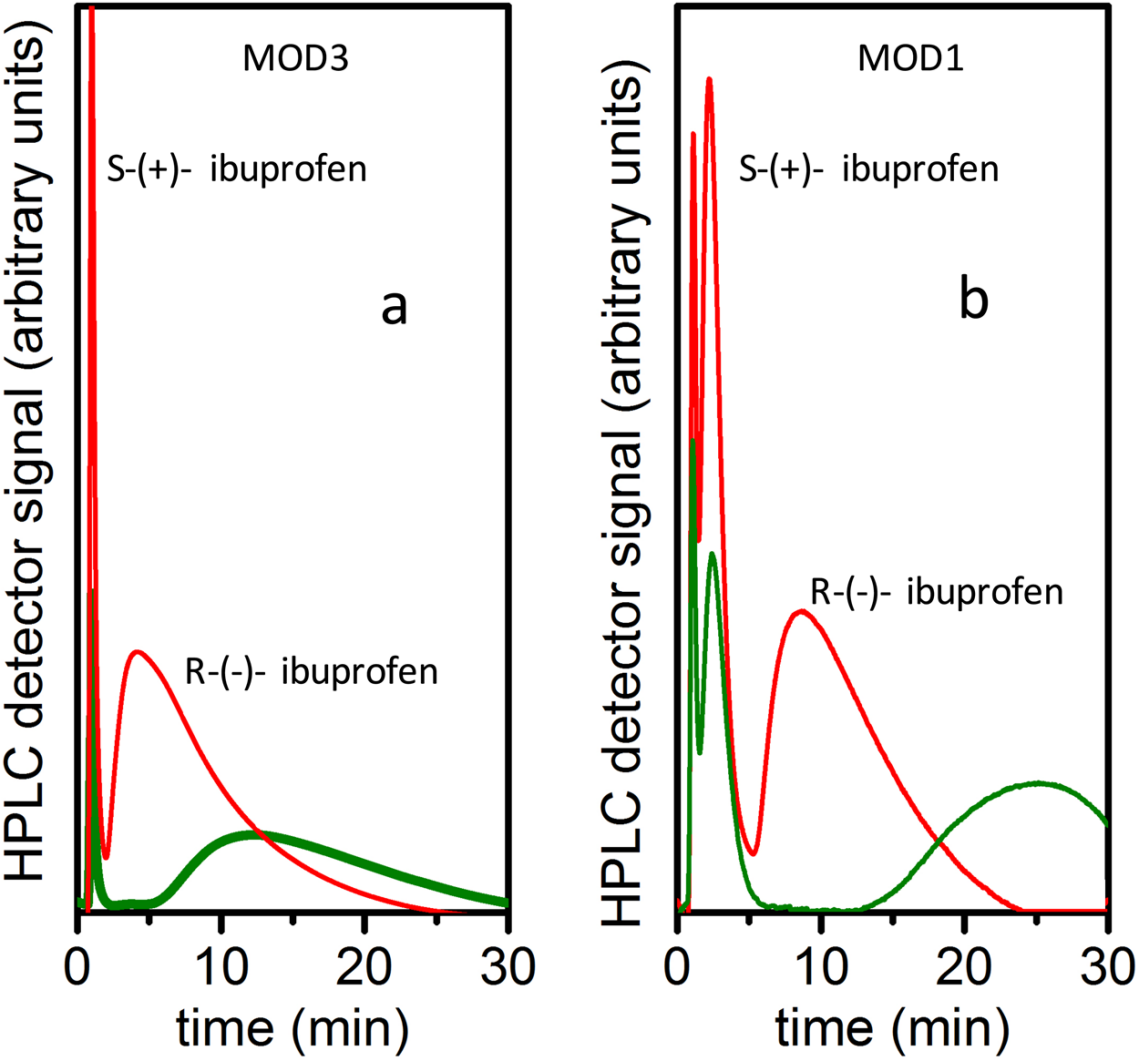
(**a**) HPLC curves for experiments show separation results of racemic ibuprofen solution with (S)-(+)-2-pyrrolidinemethanol -modified 3D GNS stationary phase (50:50 vol. % mixture of MOD3 3D GNS and graphite powder). Red curve: 2.5 mg/ml solution of racemic ibuprofen in hexane; green curve: 0.8 mg/ml solution of racemic ibuprofen in hexane. (**b**) HPLC curves for experiments with TCNEO-modified 3D GNS stationary phase (MOD1 3D GNS mixed with graphite powder). Red curve: 0.8 mg/ml solution of racemic ibuprofen in hexane; green curve: 0.2 mg/ml solution of racemic ibuprofen in hexane.

The results of HPLC separation of commercial racemic ibuprofen using (S)-(+)-2- pyrrolidinemethanol-modified 3D GNS stationary phase (MOD3) are shown in Fig. 2a. For this experiment, the HPLC parameters were: injection volume: 50 μl, draw speed: 200 μl/min, eject speed: 200 μl/min, wavelength: 254 nm, pressure 7.45 bar. As one can see (Fig. 2a), for solutions of racemic ibuprofen in hexane, (concentrations below 1 mg/ml), baseline separation of enantiomers was achieved. For ibuprofen (0.8 mg/ml), the 50:50 vol. % mixture of MOD3 3D GNS and graphite powder stationary phase demonstrated selectivity^42^ α ~ 12 and retention factors^42^ *κ* ~ 1 for S-(+)-ibuprofen and *κ*~15 for R-(−)-ibuprofen.

After successful separation experiments on the column filled with (S)-(+)-2-pyrrolidinemethanol-modified 3D GNS (MOD3), where efficient separation was expected, we decided to test separation abilities of 3D GNS material after the first step of modification, TCNEO-modified 3D GNS (MOD1). This experiment was based on theoretical suggestions concerning chiral separation abilities of graphene^36,38^ and on common knowledge of structural asymmetry of 3D GNS with attached carbonyl ylide groups.

The unique chemical stability of TCNEO attachment to 3D GNS is due to covalent C-C bonding^43^. The ultimate proof of the stability of this attachment comes directly from the process of fabrication of MOD3: the modification steps between MOD1 and MOD3 (see Methods for details) include processing of MOD1 3D GNS material in water solution of H_2_SO_4_ (at temperatures above 100 °C), and in methylene chloride. Both of these treatments are unable to break the C-C bonds and remove the attached carbonyl ylide from 3D GNS surfaces. During functionalization, the MOD1 material passes through stages of environmental pH changing in the range from 2 to 10. In ambient conditions, standard organic solvents like methanol, acetone, or acetonitrile do not result in any damage to MOD1/MOD3 material; they are used to clean these materials after the functionalization reaction (see Methods for details). To illustrate this, we performed the test where pipette MOD1-column was initially flushed with methanol and secondly with hexane. After flushing with solvents, separation of ibuprofen with this column was repeated. Data showed that separation did take place, demonstrating stability of the TCNEO-modified 3D GNS stationary phase to methanol. Another stability test of MOD1 material in extremely low pH environment, described in the Supplementary Information, demonstrated the survival of chiral separation capability of MOD1 material after a wash in 5% aqueous solution of nitric acid (pH < 1). Such wash would deactivate any silica-based chiral column.

Results of HPLC experiments of MOD1 mixed with graphite powder (50:50 vol.% composition), after “washing” with methanol at ~1 ml/min flow for 24 hours, are shown in Fig. 2b. (HPLC parameters: injection volume: 50 μl, draw speed: 200 μl/min, eject speed: 200 μl/min, wavelength: 254 nm, pressure 6.82 bar). It is important to keep in mind that a coated-type commercial separation column can be deactivated by such an aggressive wash^3,4^.

The HPLC experiments allowed us to quantify the performance of a stationary phase made out of TCNEO-modified 3D GNS (Modification 1). We achieved baseline separation of ibuprofen enantiomers in the column filled with MOD1 material (mixed with graphite powder) for concentrations below 0.4 mg/ml at the flow rate 0.2 ml/min. For 0.8 mg/ml concentration of ibuprofen solution in hexane (baseline separation), our measurements show selectivity α ~ 4, resolution^42^ *R*_s_ ~ 0.6, retention (capacity) factors *κ* ~ 1 for S-(+)-ibuprofen and *κ* ~ 8 for R-(−)-ibuprofen.

**Figure 3:**
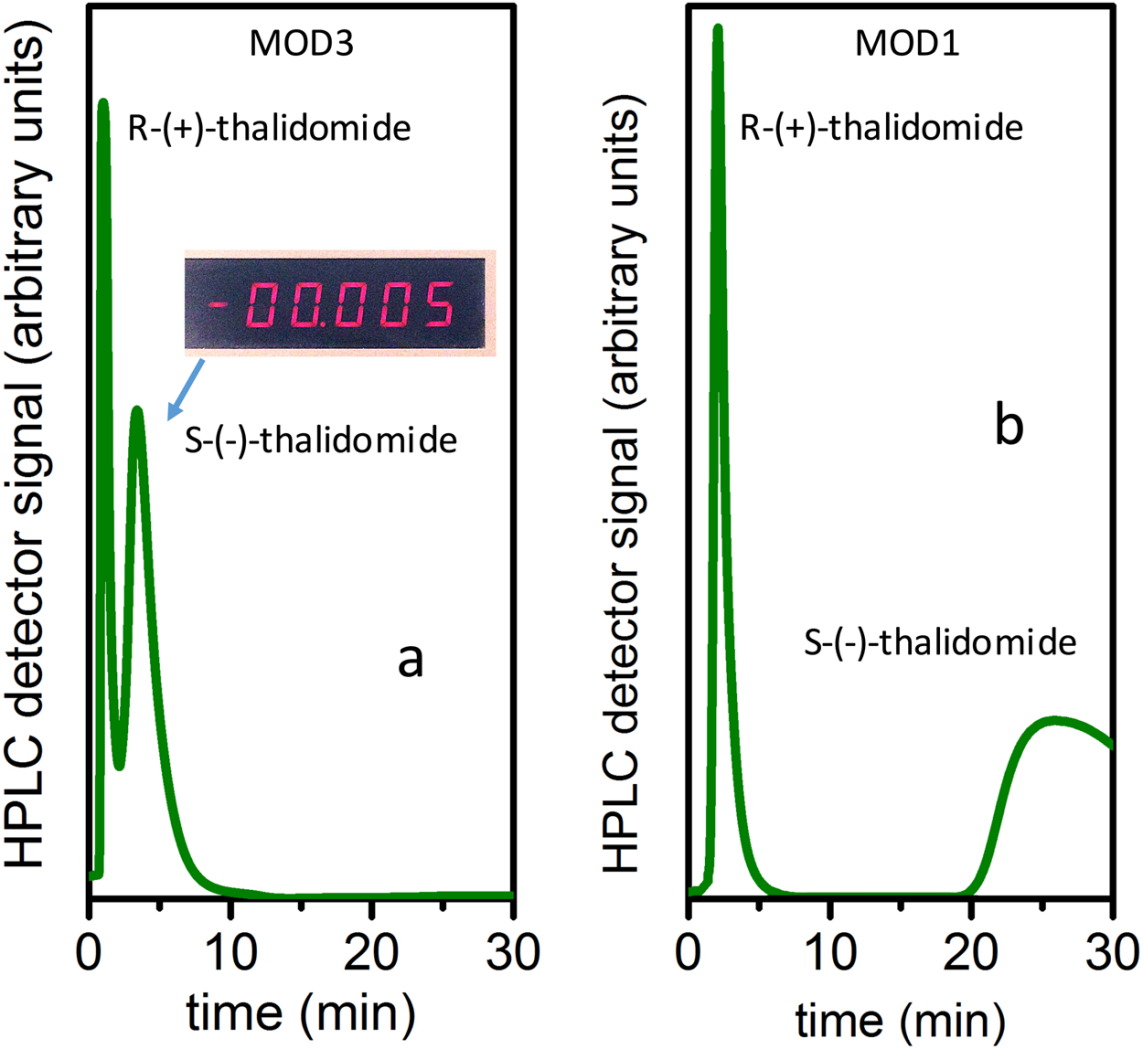
(**a**) HPLC separation curve of 0.8 mg/ml solution of racemic thalidomide in hexane with ethyl acetate, obtained with (S)-(+)-2-pyrrolidinemethanol-modified 3D GNS stationary phase (MOD3 3D GNS mixed with graphite powder). The inset shows the corresponding polarimetry result (negative angle of polarization rotation) from a flush chromatography test. (**b**) HPLC separation result (baseline separation) for 0.05 mg/ml solution of racemic thalidomide achieved in an experiment with TCNEO-modified 3D GNS stationary phase (MOD1 3D GNS mixed with graphite powder).

Thalidomide was the second racemic drug mixture we separated using the procedure discussed above. The first step was pipette separation, which gave the same sequence of separated products. Injecting racemic thalidomide, we observed the positively-rotating material being eluted first, and negatively-rotating material being eluted second from the pipette. Examples of HPLC thalidomide separation using stationary phase with MOD3 and MOD1 3D GNS are shown in Fig. 3a,b respectively. The HPLC parameters for results shown in Fig. 3a,b are: injection volume: 50 μl, draw speed: 200 μl/min, eject speed: 200 μl/min, wavelength: 254 nm. Pressure: 7 bar for 3,a, 8 bar for 3,b).

As one can see in Fig. 3a,b, in the experiment with MOD3-based 3D GNS stationary phase, for a 0.8 mg/ml thalidomide solution in hexane, we achieved selectivity α  = 3.3, resolution *R*_s_ = 0.55, retention (capacity) factors *κ* = 0.4 for R-(+)-thalidomide and *κ* = 2.7 for S-(−)-thalidomide. In the experiment with MOD1 3D GNS-based stationary phase, for the 0.05 mg/ml thalidomide solution in hexane, we received baseline separation, selectivity α = 17.5, and retention factors *κ* = 0.5 for R-(+)-thalidomide and *κ* = 22 for S-(−)-thalidomide.

## Discussion

The obtained separation results show that the fabricated MOD1 and MOD3 materials demonstrate performance comparable to commercial silica-based CSPs. The Tables 1 and 2 help to make such comparison between our 3D GNS-based materials and commercial silica-based columns (Chiralcel OJ-H and Chiralcel-OJ, Daicel Chemical Industries, Japan) utilizing cellulose as the chiral separator. Table 1 summarizes separation results for ibuprofen, and Table 2 for thalidomide.

**Table 1:** Ibuprofen separation results.

Column type	Chiracel OJ-H (silica-based/cellulose) (ref.^44^)	MOD1	MOD3
Selectivity α	0.9	4	12.5
Retention factor *κ*	not reported directly in (ref.^44^)	1 for S-(+)-ibuprofen 8 for R-(−)-ibuprofen for 0.2 mg/ml	1 for S-(+)-ibuprofen 15 for R-(−)-ibuprofen for 0.8 mg/ml
Resolution *R*_s_ or degree of separation	baseline separation (see ref.^44^)	baseline separation for 0.2 mg/ml	baseline separation for 0.8 mg/ml

**Table 2:** Thalidomide separation results.

Column	Chiracel OJ (silica-based/cellulose)(ref.^45^)	MOD1	MOD3
Selectivity α	1.54	17.5	3.3
Retention factor *κ*	9.67 for R-(+)-thalidomide (see ref.^45^)	0.5 for R-(+)-thalidomide 22 for S-(−)-thalidomide for 0.05 mg/ml	0.4 for R-(+)-thalidomide 2.7 for S-(−)-thalidomide for 0.8 mg/ml
Resolution *R*_s_ or degree of separation	15.5 (see ref.^45^)	baseline separation for 0.05 mg/ml	0.55 for 0.8 mg/ml

As one can see in Table 1, the type of modification, MOD1 or MOD3, demonstrated selectivity α of the order of 4–12.5, retention factors *κ* of the order of 1 (for S-(+)-ibuprofen) and 8–12.5 (for R-(−)-ibuprofen). Baseline chiral separation was demonstrated for ibuprofen concentrations 0.2 mg/ml (MOD1) and 0.8 mg/ml (MOD3) in hexane (with small addition of ethyl acetate for solubility of chiral compounds) used as solvent and mobile phase eluent. These results are comparable to the separation results of ibuprofen HPLC separation using commercial cellulose-based stationary phase: chiral selectivity α ~ 1 and resolution *R*_s_ ~ 0.8–1 for 2 mg/ml of ibuprofen dissolved in n-hexane, with hexane/propanol/trifluoracetic acid mobile phase^44^.

For thalidomide separation (Table 2), depending on the type of modification, our 3D GNS-based stationary phase materials demonstrate selectivity α ~ 3.3, *R*_s_ = 0.55, retention factors *κ* = 0.4 for R-(+)-thalidomide and *κ* = 2.7 for S-(−)-thalidomide for 0.8 mg/ml thalidomide solution in hexane with MOD3, and α = 17.5, *κ* = 0.5 (for R-(+)-thalidomide) and *κ* = 22 (for S-(−)-thalidomide) for the 0.05 mg/ml thalidomide solution in hexane with MOD1. These results are also comparable to the reported performance of HPLC separation of thalidomide with cellulose-based stationary phase^45,46^. For example, the authors of ref.^45^ achieved α = 1.54, *R*_s_ = 15.5, and *κ* = 9.67 (for R-thalidomide). All separation experiments were repeated at least three times to ensure reproducibility, and the above values are representative.

Table 3 illustrates the position of MOD1 and MOD3 3D GNS CSPs among other carbon-based chiral separators utilizing CNTs, GO and graphene. In discussing previous attempts of making carbon-based materials for chiral separation, it is important to mention organic polymer monoliths doped with chiral carbon nanotubes^25^. These materials were specifically designed as stationary phases for HPLC. The reported separation performance of CNT-based monoliths is reasonable: the demonstrated selectivity α ~ 2.9, reported *R*_s_ reached 2.87, and several important pharmaceuticals were reportedly baseline-separated (celiprolol, chlorpheniramine, cizolirtine, etozoline, sulconazole, miconazole, and nomifensine).

**Table 3:** Comparison of separation results for different chiral separators utilizing CNTs, GO, and graphene.

Separator type	CNT-doped organic polymer monolith (ref.^25^)	Glutamic acid-modified GO separation membrane (ref.^37^)	Alanine racemase chiral analogue-coated mesoporous carbon monolith (refs^39,40^)	Carbon nanoparticle-modified separation system with dextrin (ref.^27^)	MOD1 mesoporous 3D GNS	MOD3 Mesoporous 3D GNS
Reported selectivity α	1.1 ÷ 2.89 depending on separated compound	2 (max)		1 ÷ 2.5 depending on separated compound	17.5 (for thalidomide)	12.5 (for ibuprofen)
Reported resolution *R*_s_ or degree of separation	1.31 ÷ 2.87 depending on separated compound		90–95% separation	0.8–4.3 depending on separated compound	baseline separation for 0.05 mg/ml of thalidomide	baseline separation for 0.8 mg/ml of ibuprofen
Separated chiral compound	Celiprolol, chlorpheniramine, cizolirtine and others (ref.^25^)	D,L-DOPA (see ref.^37^)	Phenylalanine, serine, tryptophan	Sulconazole, ketoconazole, citalopram hydrochloride, nefopam hydrochloride	thalidomide, ibuprofen	ibuprofen, thalidomide
Functional role and separation technique it is designed for	Chiral stationary phase for HPLC	Membrane material for membrane filtration	Adsorbing material, adsorption	Electrode material for capillary electro kinetic chromatography	Chiral stationary phase for HPLC	Chiral stationary phase for HPLC

The majority of reported attempts to use graphene or graphene oxide for chromatographic stationary phases were not focused on chiral separation (see Table 3). Most of them were different variants of graphene or GO-doped organic polymer monoliths^41,47^. The previous reported attempts utilizing graphene or graphene oxide-based materials for chiral separation are not directly comparable with MOD1 and MOD3 materials, because they were designed and tested for other, non-HPLC separation techniques. For example, amino acid-modified graphene oxide was recently used for fabrication of separation membranes^37^. The tests of these membranes for separation of 3,4-dihydroxy-D, L-phenylalanine (D, L-DOPA) demonstrated^37^ maximum selectivity of the order of α ~ 2. The tests of mesoporous carbon monoliths for separation of amino acids (another non-HPLC approach, involving absorption and storage of one chiral component in the mesoporous material) demonstrated about 90–95% separation^39,40^. Capillary electro kinetic chromatography with carbon nanoparticle-modified chiral separation systems demonstrated α not exceeding 2.5 and typical *R*_s_ in the range of 0.8-4.3^27^. As one can see, in contrast to our results, the separation capabilities of these attempts were not competitive with commercial silica-based CSPs.

Table 3 helps to evaluate our result among currently existing CSP solutions. Standard silica-based surface-coated CSPs show high separation performance but are costly, not chemically stable, and can be deactivated by aggressive mobile phase solvents^3^. The immobilized silica gel-based CSPs work with a wide spectrum of solvents and are chemically stable, but these superior performance parameters are the result of complex multi-step fabrication processes; therefore, the price of these CSPs is high^3,4^. The reported carbon based CSPs with chiral CNTs are complex in fabrication, and have significant limitations as a result of the use of organic polymer monoliths^25,41,47^. The demonstrated chiral separators utilizing graphene or graphene oxide also have a variety of limitations: they either use weakly attached chiral molecules^29–35^ and cannot be chemically stable in many important organic solvents, or completely adsorb and block one of the enantiomers during separation^39,40^. Before our work, no reports demonstrated graphene-based CSPs for HPLC that are fully competitive with commercial immobilized CSPs. Also, for our materials, the achieved price of 3D GNS per gram of material is about $1.50. Additional expenses related to modifications increase this price per gram to about $3–4. This is about one order of magnitude less than the estimated price per gram for silica-based materials.

Further improvement for our 3D GNS-based chiral separation columns includes optimized packing of the stationary phase material. In our experiments, the asymmetry *A*_s_ (ref.^42^) of HPLC peaks corresponding to separated ibuprofen enantiomers was on the order of *A*_s_ ~2–3. The asymmetry was improved (reduced) in the experiments with thalidomide after repacking of columns down to *A*_s_ ~ 2 or better. At the same time, further improvement of packing will be necessary for the next generation of 3D GNS-based separation columns.

Summarizing, we demonstrate new variants of carbon-based stationary phases for chromatographic chiral separation, utilizing functionalized 3D graphene nanosheets. Using 3D GNS-based stationary phase materials, we successfully separated ibuprofen and thalidomide. The demonstrated separation parameters of tested 3D GNS-based HPLC columns are comparable to the current commercially available columns for chiral separation. At the same time, our 3D GNS-based stationary phases have superior chemical stability: the demonstrated TCNEO-modified 3D GNS CSPs can work with wide range of aggressive mobile phases and different organic solvents and eluents, making MOD1 CSPs competitive to immobilized CSPs^4^. Because of the simplicity of fabrication and functionalization, our materials are significantly cheaper than currently available commercial variants. Being carbon-based, our stationary phases should reveal other advantages which need to be investigated.

## Methods

### Preparation of 3-dimensional graphene nanosheets

Synthesis of 3-dimensional graphene nanosheets (3D GNS), the basis of our stationary phase materials, was done in two main steps. The first step was synthesis of graphene oxide (GO) by modified Hummers method^48–51^. Then, for fabrication of mesoporous material, we applied the Sacrificial Support Method (SSM) developed earlier in our previous works^48–50^: the synthesized GO was dispersed and fully exfoliated in the water solution by means of high power ultrasonic probe (900 kJs were delivered to 12 g of GO in 1 L of DI water for 3 hours) followed by addition of 25 g of commercial fumed silica (Cab-O-Sil, EH5, surface area ~400 m^2^ g^−1^). The mixture was further ultrasonically treated with the probe an additional hour. Viscous colloidal solution of GO-SiO_2_ was dried overnight at T = 85 °C. Dry powder was ball-milled at 400 RPM for 25 minutes followed by thermal reduction in 7at% H_2_, flow rate 100ccm, T = 850 °C, time = 1 h. After reduction hybrid of GNS-SiO_2_ was ball-milled at 400 RPM (rotations per minute) for 25 minutes. Silica support was removed by leaching out with 25 wt% HF for 24 h.

After leaching material was filtrated and washed with DI water until neutral pH was achieved. 3D GNS was dried overnight at T = 85 °C.

### Chemical functionalization

Chemical functionalization of synthesized 3D GNS was done in the three modification steps. To indicate these steps, we will use abbreviations MOD1 for the first step, MOD 2 and MOD3 for the second and the third steps, accordingly. The descriptions of modifications are the following:

#### Modification 1 (MOD1)

**Figure 4:**
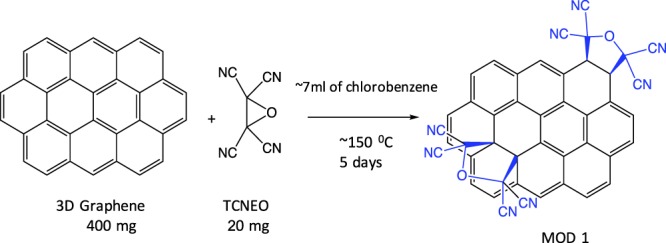
Scheme of chemical reaction of the modification 1 (MOD1): the addition of carbonyl yilide (synthesis of TCNEO-modified 3D GNS).

A mixture of 400 mg of 3D GNS and 20 mg of tetracyanoethylene oxide (TCNEO) was added to 7 ml of chlorobenzene and refluxed at a temperature of 150 °C for 24 hours. After 24 hours, a stir bar was added to the reaction mixture to begin stirring for a following 4 days (see Fig. 4). Upon completion of the reaction after 4 days, the modified graphene was filtered off, washed with acetone (3x, 5 ml), acetonitrile (3x, 5 ml), methanol (3x, 5 ml), and acetone (3x, 5 ml). Material was allowed to dry overnight and appropriate spectra was run to ensure proper modification was observed. The details of this reaction were described in the ref.^43^.

#### Modification 2 (MOD2)

**Figure 5:**
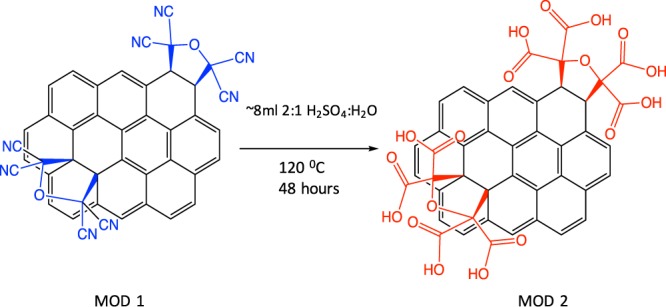
The modification step 2 (MOD2): hydrolysis of TCNEO-modified 3D GNS.

400 mg of MOD1 was added to an 8 ml mixture of water and sulfuric acid (H_2_O:H_2_SO_4_ = 1:2, volumetric) and heated to 120 °C for 2 days. Material was washed with water (3x) until pH of residual waster was approximately 6.0–7.0 (Fig. 5). Material was allowed to dry overnight and Raman, energy dispersive X-ray spectroscopy, and X-ray photoemission spectroscopy tests were run to ensure proper modification was observed (see Supplementary Information).

#### Modification 3 (MOD3)

**Figure 6:**
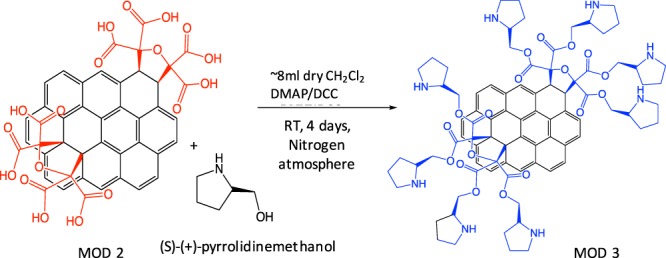
Modification 3 (MOD3): Attachment of (s)-(+)-pyrrolidinemethanol to 3D GNS after modifications 1 and 2.

Attachment of chiral molecule to 3D GNS materials after modification with carbonyl ylide followed by hydrolysis. Prior to any chemical synthesis, all glassware was dried. 400 mg of MOD2 was added to an argon dried flask (purged for five minutes). (S)-(+)- pyrrolidinemethanol (1.656 mM, 0.1675 g) and 4-Dimethylaminopyridine (DMAP, 0.442 mM, 0.0539 g) were added to flask along with 5 ml of dry CH_2_Cl_2_ (while argon atmosphere was continually maintained). Being placed on ice, N, N’-Dicyclohexylcarbodiimide (DCC) (0.6072 mM, 0.1243 g) was added drop-wise over 5 minutes to dry CH_2_Cl_2_. Then, the solutions from both flasks were combined followed by addition of 2 more ml’s of dry CH_2_Cl_2_. The MOD2 and solvents were stirred for 3 days at room temperature. The obtained solid material was washed with water (1x), Methanol (5x), Acetonitrile (3x) and acetone (3x) on a filter and dried overnight. Finally, characterization tests were run to ensure proper modification was observed (Fig. 6).

#### Reaction Yields

Modification 1: the yield of modified product was around 90%, estimated by comparing the amounts of TCNEO in solution before and after modification.

Modification 2: according to literature data, similar conditions of reaction of hydrolysis of the CN group lead to a 82–96% yield of corresponding acid depending on the structure of the hydrolyzed compound^52,53^_._ Our rough estimated yield values for this reaction are in agreement with values reported in the literature.

Modification 3: according to literature data, the conditions of Steglich esterification (we used similar conditions for our Modification 3) lead to a yield of about 95%^54^.

A more detailed description of characterization techniques we used in this work, is provided in the Supplementary Information.

## Electronic supplementary material

Supplementary Information
