# Supplementary material for: Surface-modified three-dimensional graphene nanosheets as a stationary phase for chromatographic separation of chiral drugs
Source: Sci Rep. 2018 Oct 3;8:14747. doi: 10.1038/s41598-018-33075-w (PMC6170404; doi:10.1038/s41598-018-33075-w)
Supplement: Supplementary file 1 — Supplementary Information [file 41598_2018_33075_MOESM1_ESM.pdf]

Supplementary Information

Surface-modified three-dimensional graphene nanosheets as a stationary phase for chromatographic separation of chiral drugs

Lindsay Candelaria[1], Liliya Frolova[2], Brian Kowalski[1], Kateryna Artyushkova[3], Alexey Serov[3,4,*], and Nikolai G. Kalugin[1,*]

[1]Department of Materials and Metallurgical Engineering, New Mexico Tech, Socorro NM 87801, USA
[2]Department of Chemistry, New Mexico Tech, Socorro NM 87801, USA
[3]Department of Biological and Chemical Engineering, University of New Mexico, Albuquerque NM 87131, USA
[4] Pajarito Powder, LLC, 3600 Osuna Rd NE, Suite 309, Albuquerque, NM 87109, USA

[*]Corresponding authors: nikolai.kalugin@nmt.edu, aserov@pajaritopowder.com

Characterization methods and measurement techniques.

The morphology, purity, and composition of the synthesized material were studied by scanning electron microscopy (SEM) using a Hitachi S-5200 with an accelerating voltage of 2.0 kV at 15 μA , transmission electron microscopy (TEM) using a JEOL 2010F microscope with an accelerating voltage of 200 keV and a current of 190μA, and X-ray photoelectron spectroscopy (XPS) utilizing a Kratos Analytical AXIS Ultra DLD Imaging System with an Al K-alpha X-ray source operating at 225 W. Structural properties were also characterized using Raman spectroscopy. Raman microscopy and photoluminescence measurements were performed using a Jobyn Yvon LabRam Aramis grating spectrometer equipped with a CCD camera. The second harmonic radiation of a diode-pumped Nd:YAG laser (523 nm wavelength, 5 mW power, CW regime, spot size ~3μm). The modified 3D GNS material was packed into a column housing with diameter of material chamber 6 mm and length 8.5 cm. The chiral separation capabilities of material were tested using Agilent 1200 series high performance liquid chromatography (HPLC) device equipped with G1314B wavelength detector and 1260FLD fluorescence detector using the radiation wavelength 254 nm. The results of chiral separation we controlled using optical polarimetry with help of Rudolph Research Analytical Autopol III polarimeter using light at the sodium emission line at the wavelength of 589 nm. The purity of separated chiral compounds was checked using Bruker Avance III 400 MHz NMR spectrometer. We also used energy dispersive X-ray spectroscopy (EDX) for monitoring of residual impurities, and for the verification of chemical modification of our graphene samples before and after chemical treatment. For this purpose, we used the Hitachi S-3200N scanning electron microscope equipped with Thermo Electron LN-Cooled EDX detector with spectral resolution 134 eV.

Material morphology

The morphology of synthesized 3D-GNS is shown in the Figure SI1. The material has a highly defective nature after removal of silica support. The morphology is similar to previously observed in the materials made by similar method (Refs. 48-50 of the main text).

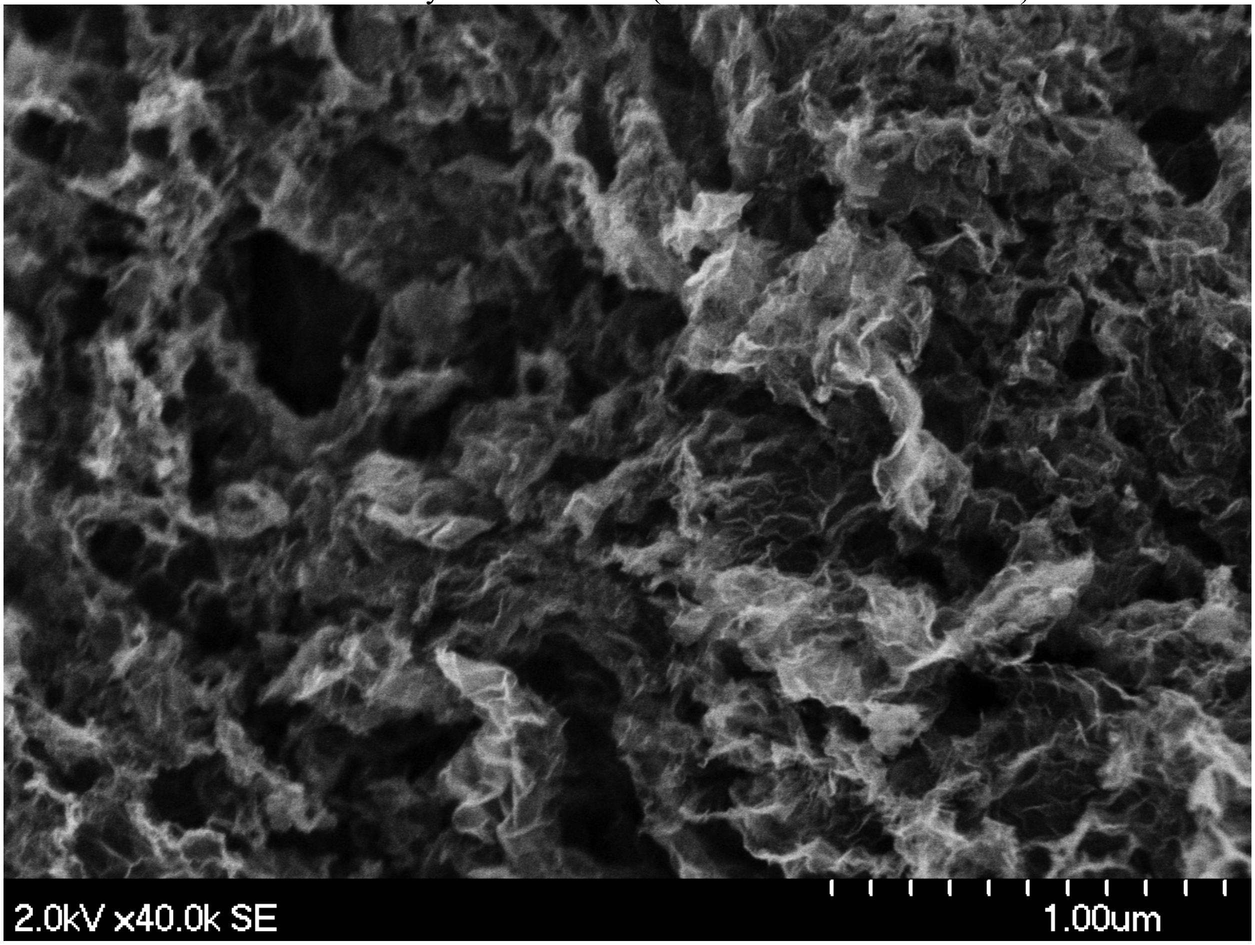

Figure SI1. Scanning Electron Microscopy image of synthesized 3D GNS material.

EDX and XPS analysis of modified 3D GNS.

The method of 3D GNS fabrication, used in our work, leads to the situation when the material before functionalization already contains chemical elements which may bind TCNEO and other functionalization chemicals. Functionalization leads to further changes in carbon, nitrogen and oxygen contents at the level comparable to the natural level of composition inhomogeneity in the initial 3D GNS. The EDX tests did not provide the statistically sufficient information about increase or decrease of quantities of chemical elements after functionalization reactions.

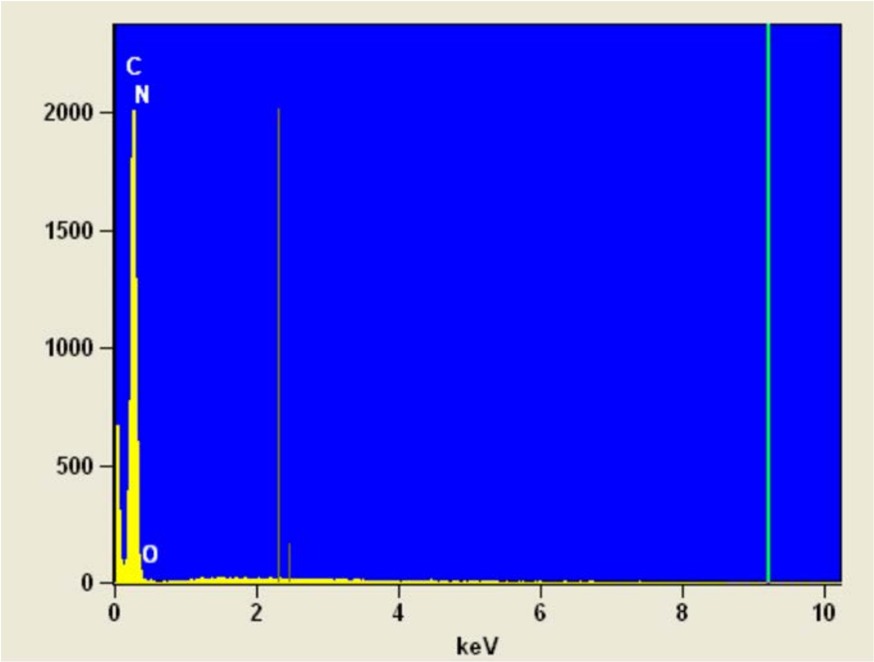

Figure SI2. The energy-dispersive X-ray spectroscopy (EDX) survey spectrum collected from the lateral size of ~3000 μm^2 area of 3D GNS sample substrate after the chemical modification 3, taken with the electron beam energy of 25 keV.

The energy-dispersive X-ray spectroscopy (EDX) spectra collected from the lateral size of ~3000 μm^2 area of 3D GNS sample substrate after the chemical modification 3, taken with the electron beam energy of 25 keV, are shown in the Figure SI2. The EDX spectrum of our fully-modified 3D GNS sample is practically identical to typical EDX spectra of 3D GNS before modifications. The EDX spectra show three dominant lines: carbon (C K-line at 0.246 keV[SI1]), oxygen (O K-line at 0.515 keV[SI1]), and nitrogen (N K-line at 0.384 keV[SI1]). EDX spectra of these samples show that the 3D GNS core material provides major contribution to EDX signals, and the modifications do not provide statistically-sufficient changes to the intensities of observed EDX lines.

The results of XPS characterization (summarized in the Table SI1) of neat 3D GNS indicate the dominant presence of carbon (above 90% of contents), 6.8% of oxygen, and 1.8 % of nitrogen. In modified samples, XPS spectra reveal clearly increased quantities of nitrogen in the samples after modification with TCNEO: the monitoring of relative intensities of C1s (XPS band location around 285 eV[SI2] and N1s XPS lines (around 400 eV[SI2]) show a notable increase in nitrogen concentration from ~1.8% to ~3.9% after the Modification 1 (TCNEO modification, MOD1), and a return to an intermediate value of ~2-3% for nitrogen after the following hydrolysis (Modification 2, MOD2).

Table SI1. Concentrations of carbon, oxygen, nitrogen, and the distribution of observed variants of carbon bonding in 3D GNS samples before and after modifications, retrieved from XPS spectra.

Sample	C 1s %	N 1s %	O 1s %	C gr %	C* %	C-N %/C-O %		C=O %	COOH %	shake %
neat 3D GNS	91.4	1.8	6.8	76.1	6.0	6.3	5.0	2.1	2.1	2.3
MOD2	91.8	1.6	6.5	86.2	0.1	3.7	3.1	2.2	1.7	3.0
MOD3	94.4	2.9	2.7	85.3	6.0	2.2	2.9	1.2	1.1	1.3

The high-resolution XPS spectra of C1s peak[SI1] are shown in the Figure SI3. The main peak due to graphitic carbon at 284.4 eV is larger for both functionalized samples. Different types of carbon oxides observed at 287 eV (C-O), 288 eV (C=O), 289 eV (COOH) also cause a peak due to secondary carbon at 285.5 eV. The total amount of surface oxides is smaller for functionalized samples. The peak due to nitrogen doped carbon is also observed at 286-287 eV.

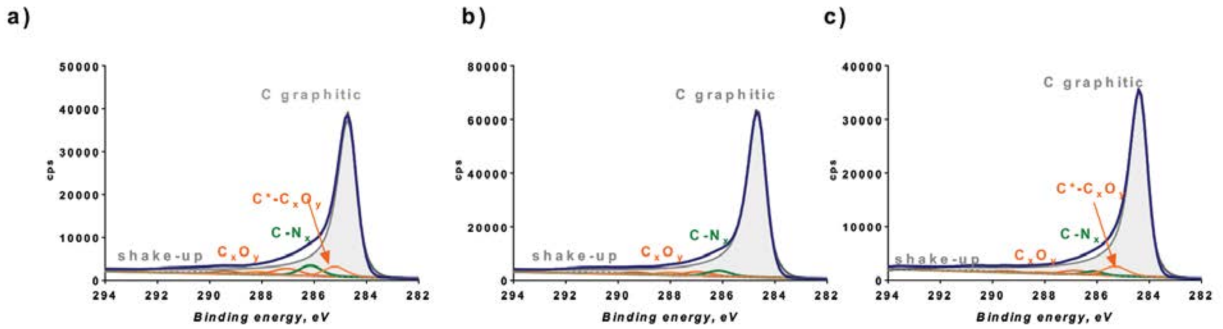

Figure SI3. The high resolution XPS spectra of C1s peak for a) 3D GNS before modifications, b) 3D GNS after TCNEO modification and following hydrolysis (MOD2), and c) 3D GNS after Modification 3 (MOD3 material).

Raman spectroscopy results

Raman spectroscopy provided the most informative feedback in characterization of 3D GNS and monitoring of modifications introduced to 3D GNS at different stages of chemical treatment. For a better adjustment of reaction parameters and observation capabilities of modification-introduced changes, we performed the same set of reactions with a sample of highly ordered pyrolytic graphite (HOPG, SPI-1 of SPI Supplies)

The results of Raman measurements of one of the HOPG samples before and after different stages of chemical treatments are shown the Figure SI4. The Raman spectra of untreated graphite (see the bottom black curve in Fig.SI4) show the typical set of major lines: G-line at 1582 cm^{-1}, and the 2D line at about 2700 cm^{-1}, and the 2D’ line at 3239 cm^{-1} (Refs.SI3-SI5). After chemical treatment of our sample with TCNEO (modification 1, corresponds to the red curve marked as MOD1 in the Fig.SI4), the Raman spectra demonstrated significant modifications. The overall spectrum shows the appearance of a significant fluorescent background, and the appearance of distinct line at 2231 cm^{-1}. This line is the well-known

signature of C-N stretching modes, which indicates that the attached (in Modification 1) structure contains cyano-groups (Ref.43 of the main text). The observed fluorescent background indicates significant modification of electron band structure in TCNEO-functionalized highly ordered pyrolytic graphite (HOPF) surface.

The second modification (MOD2), the hydrolysis, leads to the replacement of N atoms by OH groups. The Raman spectra after Modification 2 show the disappearance of C-N line, and almost complete disappearance of fluorescence (see Fig. SI4, blue curve marked MOD2). As one can see, modification 2 definitely happens, and changes the properties of HOPG-based material again.

Finally, the Modification 3- the attachment of (S)-(+)-pyrrolidinemethanol, modifies Raman spectra again. After this modification (the green curve marked MOD3 in the Fig.SI4), the spectra show the appearance of a higher fluorescence signal, and the lines corresponding to vibrations of attached pyrrolidinemethanol molecules, in particular at 320 cm^{-1}, 383 cm^{-1} (C-C-C bending mode), 501 cm^{-1} (CO torsion), 629 cm^{-1} (ring deformation), 720 cm^{-1} (NH out of plane bending), and at 1020 cm^{-1} (CO stretching mode)[SI6]. Overall, the Raman spectra indicate the successful modification of HOPG at every stage of functionalization process.

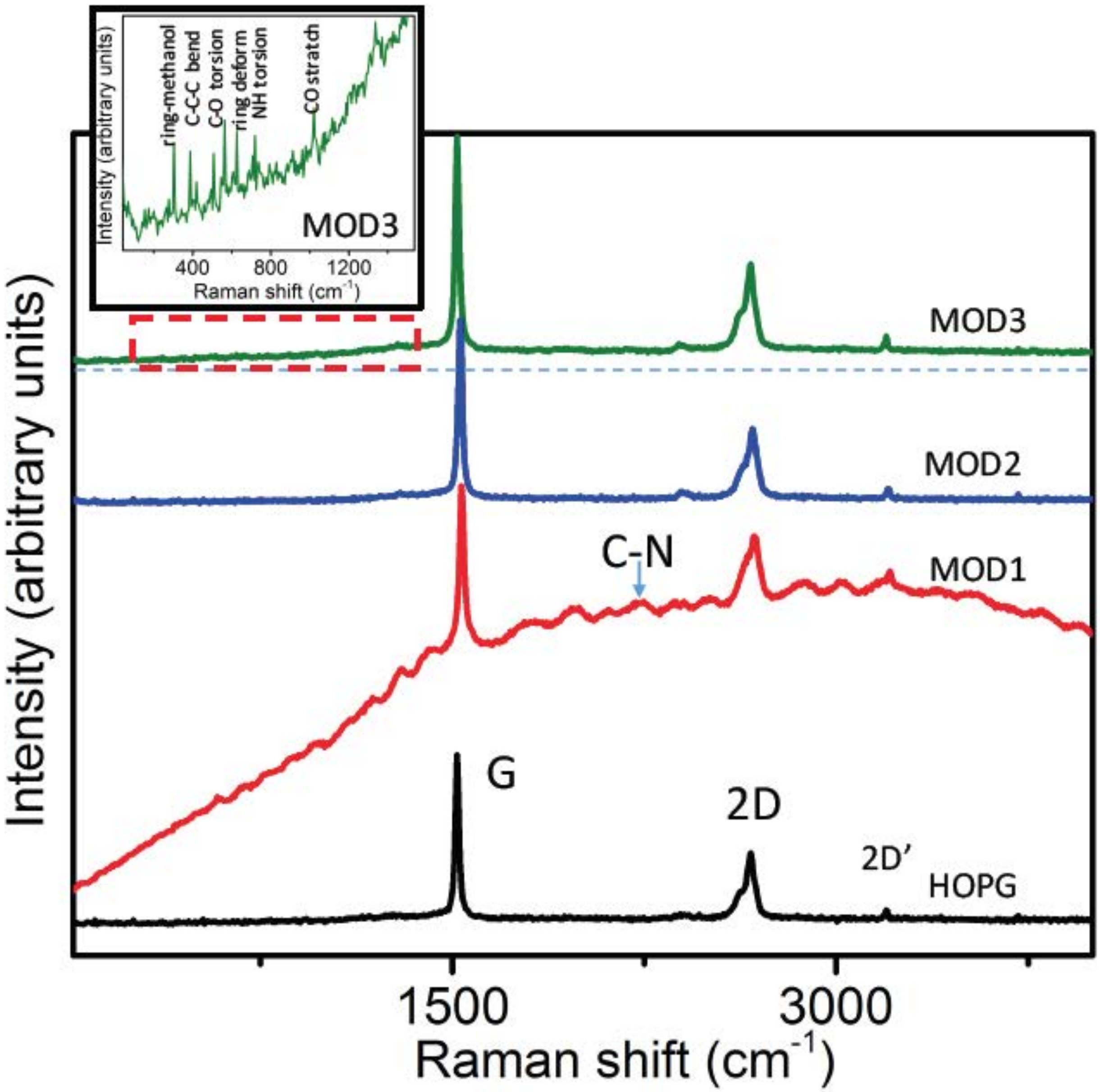

Figure SI4. (Color online) Raman spectra of highly ordered pyrolytic graphite (HOPG) at the different stages of modification. The spectra of a graphite sample taken before (bottom curve, black color, marked HOPG) and after different stages of modifications marked as MOD1, MOD2, and MOD3 accordingly (see the article text for details). For the discussion of new lines appearing in the spectrum after modification 3 (shown in the inset) see the text. The excitation wavelength λ_{exc}=532 nm.

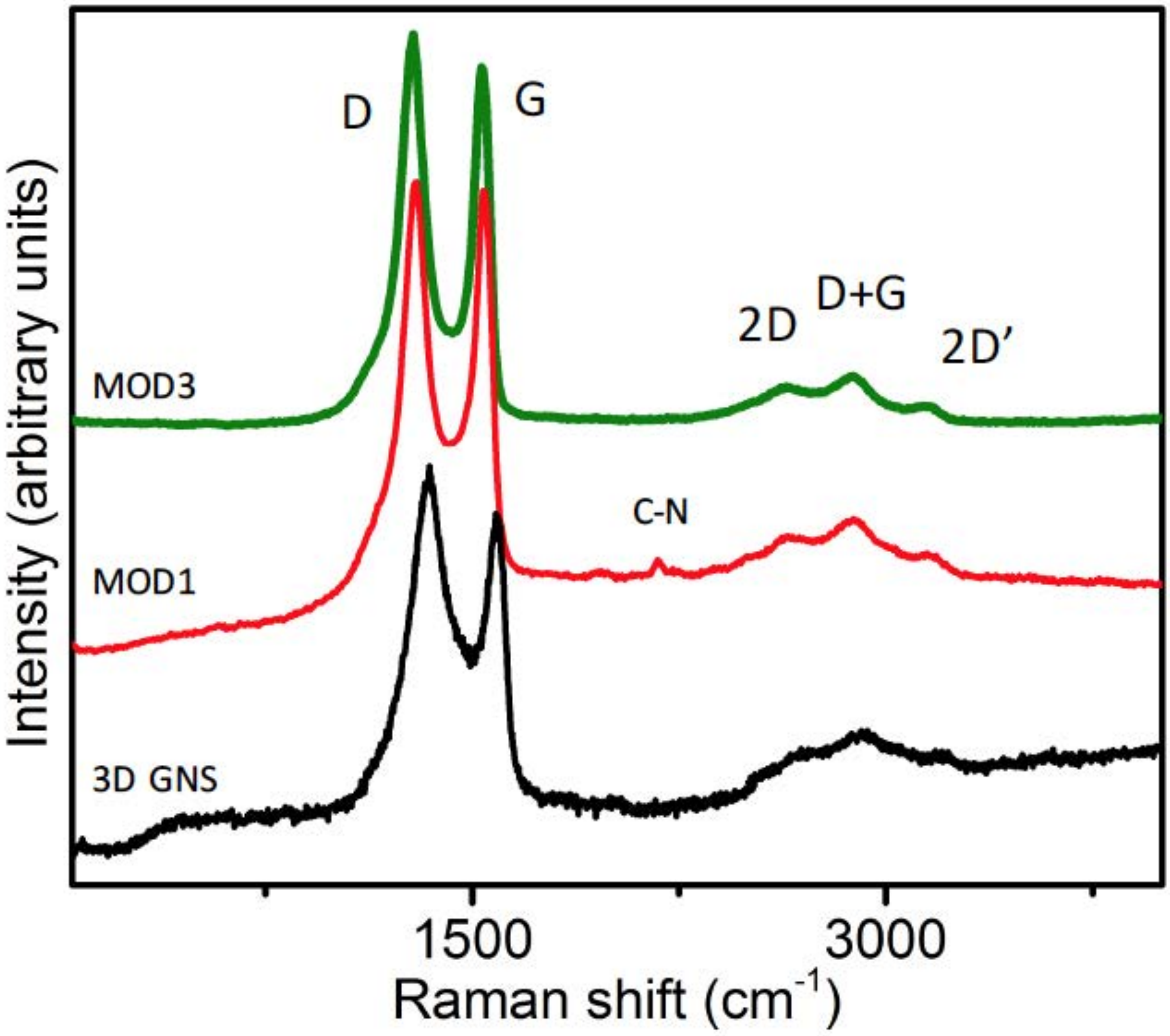

Figure SI5. Raman spectra of 3D GNS before (black curve, marked 3D GNS) and after modification with TCNEO (modification 1, marked MOD1, red curve), and after following hydrolysis and attachment of (S)-(+)-2-pyrrolidinemethanol (modification 3, MOD3, green curve). Excitation wavelength: 532 nm.

The Raman spectra of unmodified 3D GNS (Fig. SI5, black curve, marked 3D GNS) also demonstrate the major carbon Raman bands: D, G, 2D, 2D', as well as the combination D+G band. The band at 1340-1350 cm^{-1} (D-band)[SI3-SI5, SI7] occurs in Raman spectra because of reduced symmetry of material (comparing to uniform HOPG or graphene) due to defects and crumpling of graphene sheets. The rather broad band G around 1582 cm^{-1} corresponds to the E_{2g} carbon vibration mode. The high frequency bands near 2900-3000 cm^{-1} are the second order and combination lines located close to 2D, 2D', and D+G combination frequencies[SI5] (see Fig.SI5).

Modifications of 3D GNS lead to the results similar to that for HOPG. The modification 1 (attachment of carbonyl ylide) leads to the appearance of the C-N line, and to some fluorescence (red curve MOD1 in the Fig.SI5), the modifications 2 and 3 remove the C-N line, add (barely detectable in the case of 3D GNS) lines corresponding to the attached (S)-(+)-

pyrrolidinemethanol (green curve MOD3 in the Fig. SI5), and change fluorescent background in Raman spectra. Overall, the behavior of changes in Raman spectra is rather similar to that for HOPG.

Flush chromatography and polarimetry analysis.

The construction of flash chromatography pipette column is shown in the Figure SI6. The glass pipettes (inner diameter of ~8mm) were filled with stationary phase material (modified 3D GNS mixed with unmodified graphite powder). The solutions of ibuprofen and thalidomide in hexane with addition of ethylacetate (100 μl of ethylacetate per 1 ml of hexane for initial dissolving of ibuprofen and 200 μl of ethylacetate per 1ml of hexane for initial dissolving of thalidomide, accordingly), were loaded from the top of pipette. The transport of solutions through pipettes was supported by nitrogen flow with pressure slightly above 1 atm. The product of separation was collected after equal time intervals starting from the beginning of separation process.

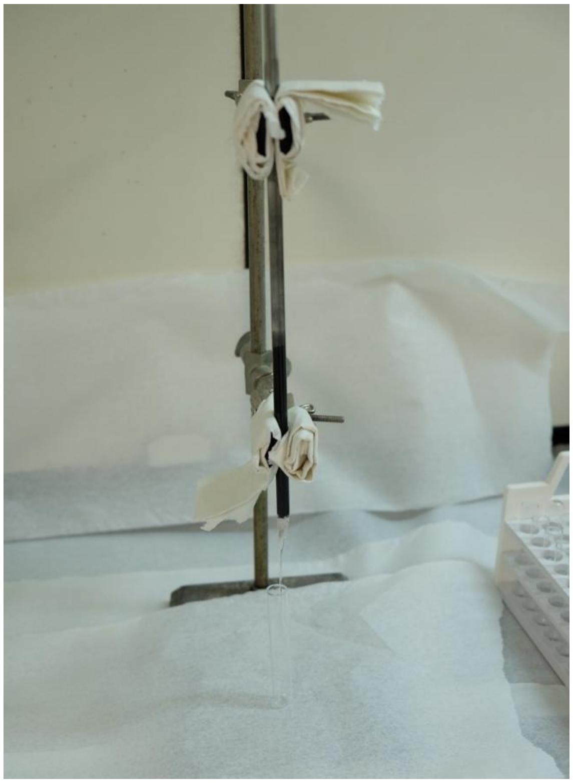

Figure SI6. The construction of pipette column. A cotton ball at the bottom of pipette was used as a stopper for our carbon-based separation material. The ibuprofen and thalidomide samples were loaded to the column from the top, and the fractions passed the column were collected in a drop-by-drop way at the bottom of column.

As an example of used procedure, we will describe all the steps of polarimetric test for MOD3 and MOD1 3D GNS CSPs with ibuprofen. Similar procedure was used in case of thalidomide.

In the pipette separation experiment with the MOD3-stationary phase and ibuprofen, we performed collection and separation 5 times, injecting every time 0.5 mg of racemic ibuprofen dissolved in 0.5 ml of hexane with 50 μl of ethylacetate. The visualization of ibuprofen presence in collected samples revealed two times delay periods when ibuprofen was coming out of pipette column (see Figure SI7). The first appearance of ibuprofen was shortly after injection (maximum of ibuprofen in the 3rd-6th drops from the pipette the Figure SI7, approximate drop volume 54 μl), subsequently, an observed visual gap was noted where there was a lack of ibuprofen , and finally an observed second-time interval of an ibuprofen signal was noted using flash chromatography plate visualization under UV. We repeated the flash chromatography experiment with racemic ibuprofen solution 5 times in order to collect sufficient amount of material after pipette separation for polarimeter experiments. The collected fractions of ibuprofen-hexane solution after flash chromatography separation were dried from hexane, and then dissolved in 10 ml of hexane for polarimetric measurements. The dry quantities of material after pipette separation were 4.7mg for the first fraction, and 4.2 mg for the second, delayed fraction of ibuprofen.

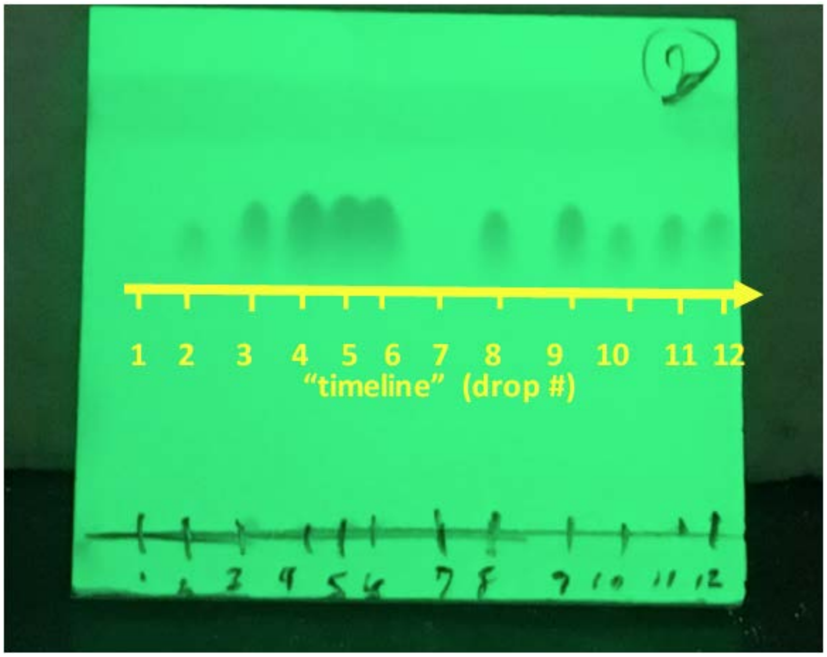

Figure SI7. Visualization of separation results of racemic ibuprofen in pipette column filled with mixture of three-steps-(S)-(+)-2-pyrrolidinemethanol modified 3D GNS (MOD3) with graphite powder (detected via quenching of fluorescence on fluorescent visualization panel). One can see the two time periods when ibuprofen comes out of column: in the drops 3-6, then pause, and then in the drops 8-12, with peak concentration in the 9th drop.

The polarimetry test of separated material (see Fig.SI8) revealed the nature of the observed two peaks (two fractions) in separation: the first one was the material demonstrating positive angle of polarization rotation (i.e. S-(+)- ibuprofen- rich material), and the second peak of concentration belongs to negative angle rotating material (i.e. R-(-)-ibuprofen-rich material).

As one can see, the 3D GNS modified with (S)-(+)-2-pyrrolidinemethanol did not cause significant delay to S-(+)-ibuprofen but slowed down propagation of R-(-)-ibuprofen. S-(+)-ibuprofen was eluted first, and the R-(-)-ibuprofen elution was delayed by the column material.

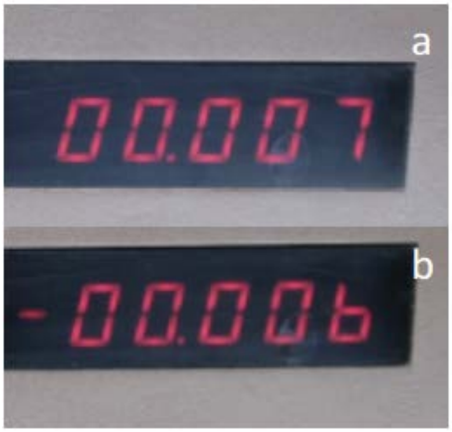

Figure SI8. Polarimetric results (polarization rotation) for the separation experiment of ibuprofen racemate in pipette column filled with (S)-(+)-2-pyrrolidinemethanol -modified 3D GNS (MOD3) mixed with graphite powder, (a)-for the first concentration peak (drops 3-6 of the Figure SI6), and (b) for the second concentration peak (drops 8-12 of the Figure SI7).

As a reference, we performed polarimetry tests of commercial ibuprofen enantiomers of comparable quantities. These results are illustrated in the Figure SI9. The observer significant difference of optical rotation between our separation results in pipette column for a racemic mixture of ibuprofen (Fig.SI8), and rotation angles demonstrated by comparable quantities of pure enantiomers (Fig. SI9), simply indicates on incomplete separation of enantiomers in our pipette separation tests.

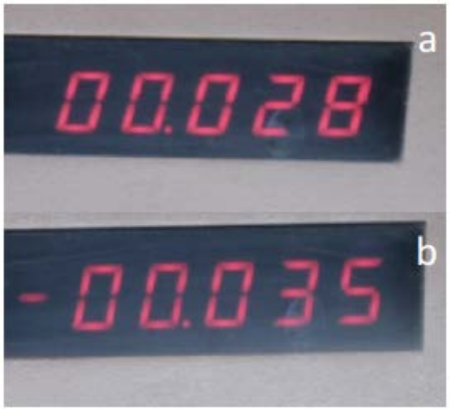

Figure SI9. The optical rotation results for (a) solution of commercial S-(+)-ibuprofen in hexane, (b) solution of commercial R-(-)-ibuprofen in hexane. Concentrations of compounds in this test: S-ibuprofen 3.8mg in 10 ml of hexane, R-ibuprofen 3.2 mg in 10ml of hexane.

The stationary phase made of TCNEO-modified 3D GNS (Modification 1) mixed with graphite powder in the same proportion as in experiments with (S)-(+)-2-pyrrolidinemethanol -modified

3D GNS, also demonstrated separation of ibuprofen enantiomers. We have found, that TCNEO-modified GNS separates ibuprofen in rather similar way (see Figure SI10), similarly to separation experiment with MOD3 stationary phase, for the MOD1 stationary phase we also observed two peaks of concentration (corresponding to the two ibuprofen enantiomers), and, again, the S-(+)-ibuprofen was propagating through stationary phase faster than R-(-)-ibuprofen. In this experiment, the first –coming fraction of ibuprofen in amount of 3.8 mg, dissolved (for polarimetry) in 10ml of hexane, demonstrated positive 0.008 deg rotation, and the second fraction, 3.2 mg of ibuprofen, dissolved in 10 ml hexane, gave negative 0.003 deg rotation of polarization. For this experiment we collected material from the sequence of 9 pipette separations, injecting ~0.05mg of racemic ibuprofen dissolved in 0.7 ml of hexane in each separation. Small observed angles of rotation instead of higher numbers (estimated magnitude of rotation for this amount of pure enantiomer is ~0.013 deg), again, indicate incomplete separation in our flush chromatography experiment.

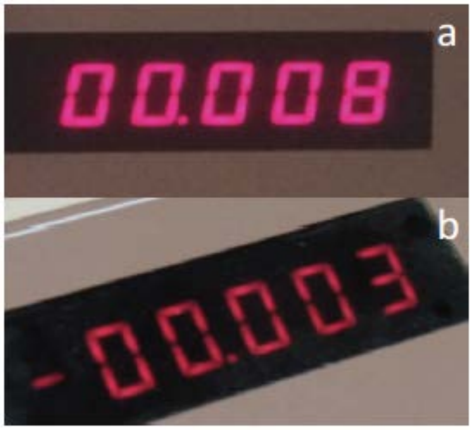

Figure SI10. Polarimetry results (polarization rotation) for the separation experiment of ibuprofen racemate in pipette column filled with TCNEO-modified 3D GNS (MOD1- material) mixed with graphite powder, (a)-for the first concentration peak, and (b) for the second concentration peak.

Stability test of MOD1 material

Silica-based chiral columns are sensitive to pH of solvents/eluents of the mobile phase. Even stable immobilized silica-based chiral columns are deactivated under extreme conditions of pH<2 or pH>8. The stability of our MOD1 material is higher. During functionalization, the MOD1 material passes through stages with pH changing in the range from 2 to 10. In our stability demonstration experiment, we show the survival of chiral separation capability of MOD1 material after a wash in 5% aqueous solution of nitric acid (pH<1). Such a wash would deactivate any silica-based chiral column.

In this test, we used 400mg of MOD1 3D GNS material mixed with graphite powder (50:50 vol%), and packed it into two pipettes of equal size and geometry (glass pipettes identical to the one shown in Figure SI6). One pipette (with 200mg of MOD1-graphite mixture) was used as reference, and the other 200mg MOD1-graphite mixture-filled pipette was treated with 5% aqueous solution of HNO_3, such that the CSP MOD1 material was left completely filled with nitric acid for 15 minutes. Then, the nitric acid was washed out from tbe material with water. We kept the water running through the pipette until the pH of the outgoing water became completely

neutral. Next, the material in the pipette was washed with 7 ml of acetone, then with hexane, and then dried by compressed air. Then, both pipette columns were saturated with hexanet and used for separation of a racemic mixture of ibuprofen. In these separation experiments, we used 0.2mg/ml solution of racemic ibuprofen in hexane.

Using the separation procedure identical to that described above, in the reference pipette column (not treated with nitric acid) we observed, as expected, two separation peaks. After four 0.5 ml loads, we collected 1.7 mg of separated material in the first peak, and 1.5 mg of separated material in the second peak. For polarimetry, we dissolved the collected fractions in 9 ml of hexane each, in order to have enough volume for complete filling of the polarimeter cell. The polarimetry results for these fractions are shown in Figure SI11, a and b. As one can see, we observed the predictable chiral separation of ibuprofen, similar to our previous observations.

The same procedure was repeated with the second MOD1 material-filled pipette column, which was treated with nitric acid. With this column, we ran two 0.5ml loads of ibuprofen solution, also observed two concentration peaks, and collected 1.2 mg of material for the first concentration peak, and the same amount for the second peak. For polarimetry, we used a smaller volume of hexane (7 ml) to dissolve collected fractions, because the first experiment demonstrated that this volume should be sufficient for the polarimetry cell. The results of optical rotation measurements after separation by nitric acid-treated MOD1-based stationary phase material are shown in Figure SI11, c, and d. Very clearly, the separation was successful, the column passed the HNO_3-test, and was not deactivated by the very aggressive (pH<1) acid.

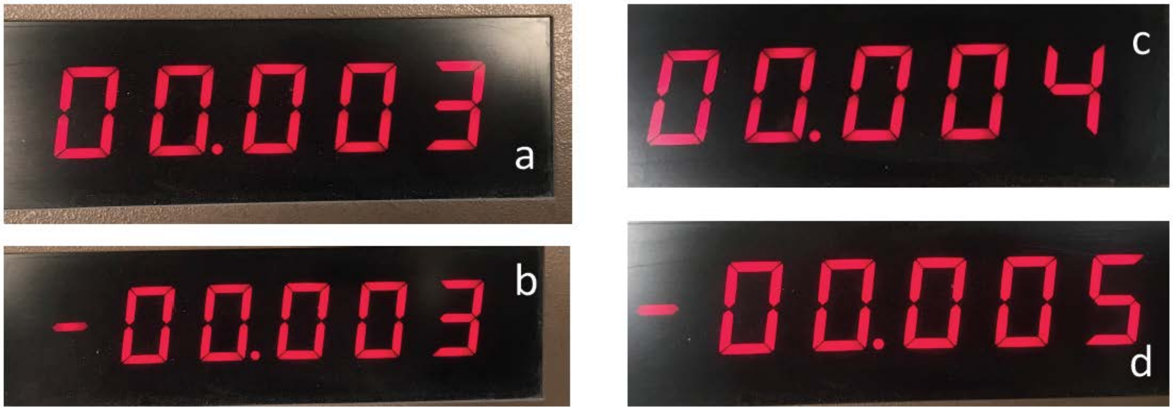

Figure SI11. Polarimetry results (polarization rotation) for the separation experiment of ibuprofen racemate. (a),(b): reference MOD1-material filled pipette, (c),(d): for the MOD1-filled pipette after treatment of CSP material by 5% aqueous solution of nitric acid (pH<1). Here, (a) and (c) correspond to the first concentration peak, and (b) and (d) to the second concentration peak (see the text for details).

HPLC experiments

In HPLC experiments, where we used standard HPLC chromatographic setup (Agilent 1200) allowed us to make quantitative evaluation of separation ability of our materials.
With this standard HPLC setup, we used the housing of a commercial column filled with our materials. The HPLC column attached to the chromatograph is shown in the Figure SI12,a. The photo of the entire HPLC setup during separation experiments is shown in the Figure SI12,b. The

preliminary tests of HPLC columns filled with modified 3D GNS material revealed significant problems related to self-packing of our 3D GNS material. We solved these problems using mixture 3D GNS materials with unmodified commercial graphite powder. We packed the column with 500mg of 50:50% volume amounts mixture of 100mg of modified 3D GNS and 400mg of commercial graphite powder with particle size <150 µm (Sigma Aldrich 496588). Use of such mixture with graphite powder significantly reduced density fluctuations of stationary phase material caused by flow of liquid.

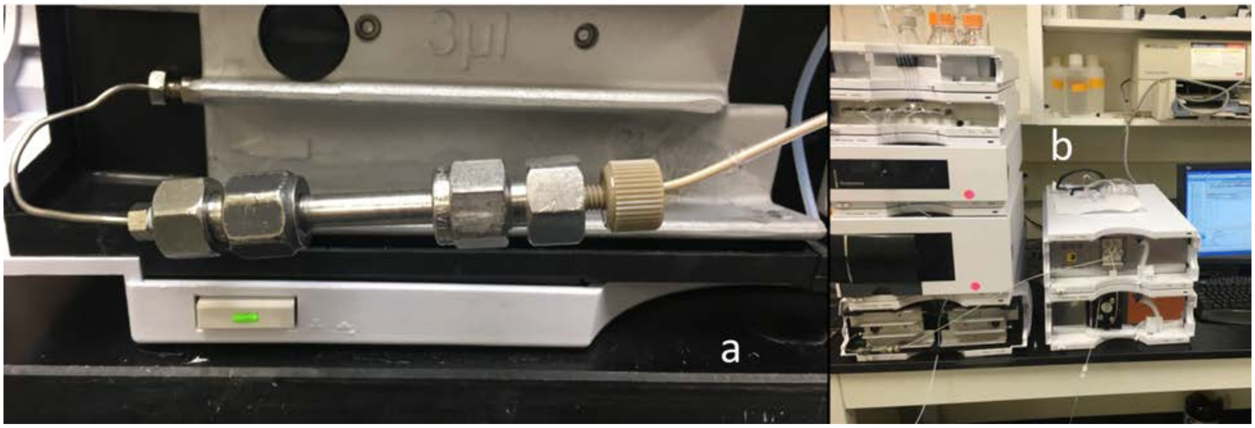

Figure SI12. HPLC column (a) and the HPLC chromatograph with attached 3D GNS-based stationary phase-filled column during separation experiment (b).

The same proportions of 3D GNS material and graphite powder were used in our pipette experiments. The body of a commercial separation column we used in our HPLC experiment had the material chamber with diameter 6 mm and length 85 mm. A standard porous metallic membrane was used for preventing migration of particles of stationary phase from leaking and blocking the exit/output tube tubes.

The reference point in our separation experiments was the test of unmodified 3D GNS material. This material, mixed 50:50% volume amounts with graphite powder (for avoiding self-packing), did not demonstrate any separation of enantiomers in pipette experiment. The fact of no separation has been confirmed in HPLC experiment (see Figure SI13): both S and R ibuprofen (dissolved in hexane) propagate through the column with unmodified material with similar time delay. The racemic mixture of S-(+)- and R-(-)- ibuprofen was giving a single peak in chromatogram, showing no separation.

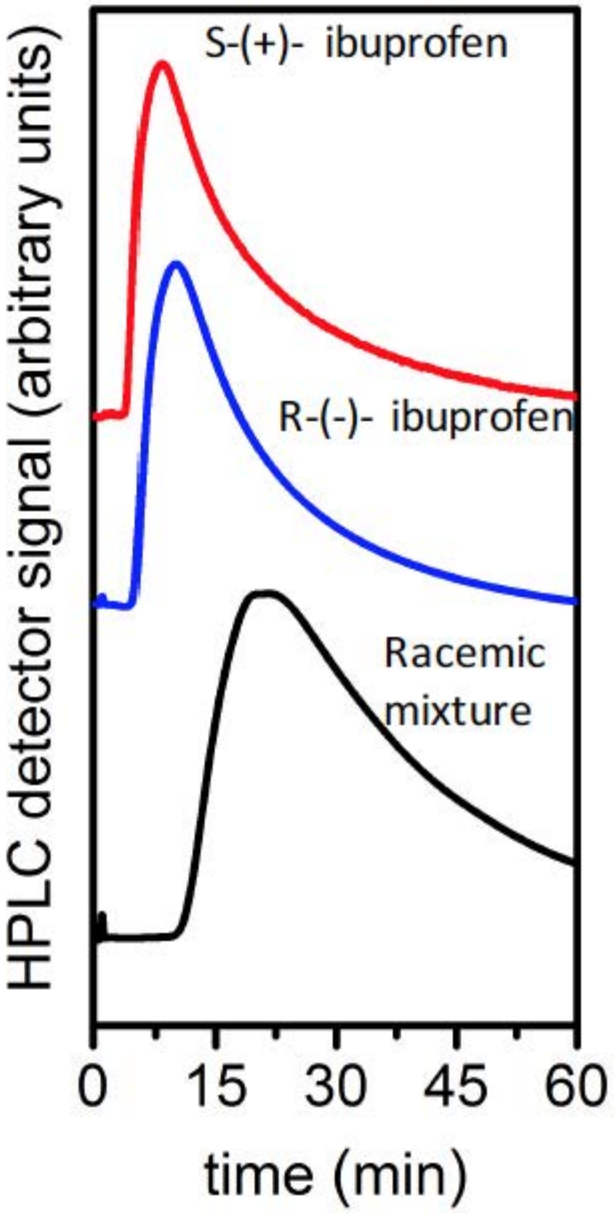

Figure SI13. The HPLC chromatograms of commercial S-(+)-ibuprofen (2.5 mg/ml solution of S-ibuprofen in hexane) and R-(-)-ibuprofen (similar concentration as for S-(+)-ibuprofen), and of racemic ibuprofen in the experiment with column filled with unmodified 3D GNS mixed with graphite powder. HPLC parameters: injection volume: 50 µl, draw speed: 200 µl/min, eject speed: 200 µl/min, detection wavelength: 254 nm, pump pressure: 10.34 bar.

Separation experiments with modified 3D GNS gave very different results. We expected the highest separation efficiency from the 3D GNS material which passed all three stages of modification: the material with attached chiral molecule of (S)-(+)-2-pyrrolidinemethanol. The idea of making this material is rather standard, this material is conceptually similar to that for commercial chiral separation materials, for example silica particles functionalized with cyclodextrin molecules. Indeed, our separation test in pipette column, loaded with (S)-(+)-2-

pyrrolidinemethanol-modified 3D GNS mixed with graphite powder, demonstrated successful separation.

As already mentioned, the polarimetry test of separated material (see Fig.SI8) revealed the nature of the observed two peaks (two fractions) in separation: the first one was the material demonstrating positive angle of polarization rotation (i.e. S-(+)- ibuprofen- rich material), and the second peak of concentration belongs to negative angle rotating material (i.e. R-(-)-ibuprofen-rich material). As one can see in Fig.SI8, the 3D GNS modified with (S)-(+)-2-pyrrolidinemethanol did not cause significant delay to S-(+)-ibuprofen but slowed down propagation of R-(-)-ibuprofen. S-(+)-ibuprofen was coming first, and the R-(-)-ibuprofen was delayed by the material of column.

In parallel with polarimetry tests with separation of ibuprofen in the pipette column, we performed NMR measurements of the separation products. While the only possible compound which we would expect as the source of negative polarization rotation angle (Fig.SI8,b) is R-(-)-ibuprofen, the positive rotation angle (Fig. SI8,a) may come not only from S-(+)-ibuprofen, but also from the (S)-(+)-2-pyrrolidinemethanol attachment to 3D GNS, caused by its physical detachment. For NMR measurements, we used the material after polarimetry measurements, evaporated hexane out, and then re-dispersed solid materials in 0.7 ml of deuterated acetone. The NMR results show that the only compound detected is ibuprofen (see Figure SI14), i.e. none of modifying molecules were desorbed from the surface of 3D GNS stationary phase.

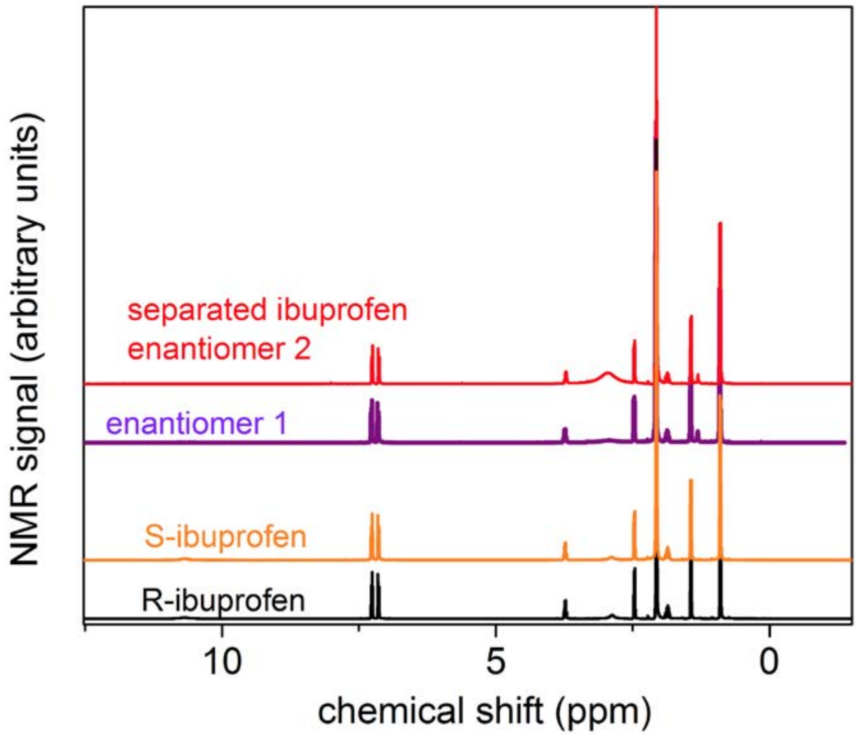

Figure SI14. The NMR probing test of separated ibuprofen (the deuterated acetone solvent signal is normalized, see text for details). The NMR spectra of commercial S-(+)- and R-(-)-ibuprofen are shown for reference.

The HPLC experiments with the column filled with (S)-(+)-2-pyrrolidinemethanol -modified 3D GNS material (our MOD3), mixed with graphite powder, confirmed our previous observations. In the experiment with commercial enantiomers, S-(+)-ibuprofen was passing the column faster than R-(-)-ibuprofen had a longer retention time (See Figure SI15).

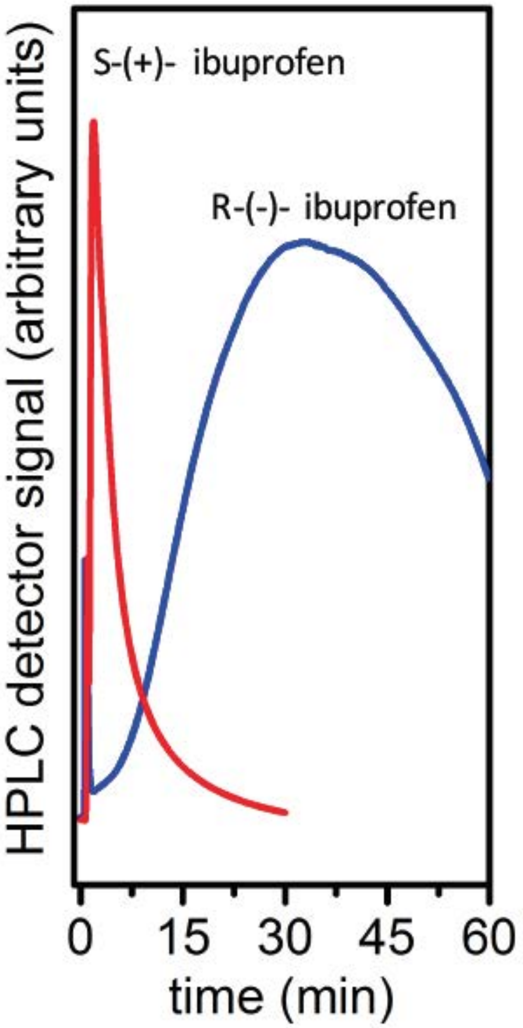

Figure SI15. The HPLC curves obtained in the experiments with commercial S-(+)- and R-(-)-ibuprofen (2.5 mg/ml solution in hexane) with column filled with (S)-(+)-2-pyrrolidinemethanol -modified 3D GNS (50:50 Vol. % mixture of MOD3 3D GNS and graphite powder). Injection volume: 50 µl, draw speed: 200 µl/min, eject Speed: 200 µl/min, wavelength: 254 nm, pressure 7.26 bar.

References

SI1. Goldstein, J *et al. Scanning Electron Microscopy and X-ray Microanalysis*, *3rd ed.* (Springer, 2003).

SI2. Watts, J.F. & Wolstenholme, J. *An Introduction to Surface Analysis by XPS and AES*, *2nd ed.* (Wiley, 2003).

SI3. Ferrari, A.C. *et al*. Raman spectrum of graphene and graphene layers. *Phys.Rev.Lett.* **97**, 187401 1-4 (2006).

SI4. Lenski, D.R. & Fuhrer, M.S. Raman and optical characterization of multilayer turbostratic graphene grown via chemical vapor deposition. *J. Appl. Phys.* **110**, 013720 1-3 (2011).

SI5. Tan, P., Dimovski, S. & Gogotsi, Y. Raman scattering of non–planar graphite: arched edges, polyhedral crystals, whiskers and cones. *Phil. Trans. R. Soc. A* **362**, 2289-2310 (2004).

SI6. Tonannavar, J., Chavan, Y.B. & Yenagi, J. (R)-(−)-2-Pyrrolidinemethanol: A combined experimental and DFT vibrational analysis of monomers, dimers and hydrogen bonding. *Spectroch. Acta A: Molec. Biomolec. Spectrosc.* **149**, 860-868 (2015).

SI7. Kalugin, N.G., Roy, A.J., Artyushkova, K. & Serov, A. Synthesis, characterization, and photoluminescence of Er_2O_3–Er_2SO_2 nanoparticles on reduced graphene oxide. *Nanotechnology* **28**, 195603 1-6 (2017).
